# Role of mitochondrial calcium uniporter‐mediated Ca^2+^ and iron accumulation in traumatic brain injury

**DOI:** 10.1111/jcmm.14206

**Published:** 2019-02-12

**Authors:** Li Zhang, Handong Wang, Xiaoming Zhou, Lei Mao, Ke Ding, Zhigang Hu

**Affiliations:** ^1^ Department of Neurosurgery Jinling Hospital, School of Medicine, Nanjing University Nanjing Jiangsu Province China

**Keywords:** Ca^2+^, iron, mitochondrial calcium uniporter, neuroprotection, traumatic brain injury

## Abstract

Previous studies have suggested that the cellular Ca^2+^ and iron homeostasis, which can be regulated by mitochondrial calcium uniporter (MCU), is associated with oxidative stress, apoptosis and many neurological diseases. However, little is known about the role of MCU‐mediated Ca^2+^ and iron accumulation in traumatic brain injury (TBI). Under physiological conditions, MCU can be inhibited by ruthenium red (RR) and activated by spermine (Sper). In the present study, we used RR and Sper to reveal the role of MCU in mouse and neuron TBI models. Our results suggested that the Ca^2+^ and iron concentrations were obviously increased after TBI. In addition, TBI models showed a significant generation of reactive oxygen species (ROS), decrease in adenosine triphosphate (ATP), deformation of mitochondria, up‐regulation of deoxyribonucleic acid (DNA) damage and increase in apoptosis. Blockage of MCU by RR prevented Ca^2+^ and iron accumulation, abated the level of oxidative stress, improved the energy supply, stabilized mitochondria, reduced DNA damage and decreased apoptosis both in vivo and in vitro. Interestingly, Sper did not increase cellular Ca^2+^ and iron concentrations, but suppressed the Ca^2+^ and iron accumulation to benefit the mice in vivo. However, Sper had no significant impact on TBI in vitro. Taken together, our data demonstrated for the first time that blockage of MCU‐mediated Ca^2+^ and iron accumulation was essential for TBI. These findings indicated that MCU could be a novel therapeutic target for treating TBI.

## 1 INTRODUCTION

Traumatic brain injury (TBI) is one of the leading causes of disability and death in modern society, resulting in high medical costs.^1^ It is defined as any head injury with traumatic aetiology, such as penetrating or blunt trauma and non‐accidental injury. The pathological process of TBI includes both primary and secondary brain injury. Although the primary brain damage is the major factor determining the patients’ outcomes, the secondary brain damage induced by multiple pathological processes, such as inflammation, cell death, apoptosis, oxidative stress and impaired calcium and iron homeostasis, provides the possibility for clinical intervention.^2^, ^3^ Despite the efforts on searching effective methods to attenuate the secondary brain injury, patients suffering with TBI always end up with poor prognosis.^4^ Therefore, new and effective strategies of treatment are urgently needed to reduce the heavy disease and economic burden.

As a second messenger, calcium (Ca^2+^) plays a key role in modulating multiple cellular physiological functions. The dynamic unbalance of Ca^2+^ in mitochondria or cytoplasm has been suggested to be associated with various physiological process including adenosine triphosphate (ATP) production, neuronal activation, cell death and apoptosis. The mitochondrial calcium uniporter (MCU) is a critical transmembrane protein that allows the passage of Ca^2+^ from cytosol to mitochondria.^5^ Under physiological conditions, MCU transports Ca^2+^ to mitochondrial matrix, retaining the homeostasis of Ca^2+^. The balance of Ca^2+^ in cell is essential for the cell's energy supply and cell survival.^6^ However, under pathological conditions, excessive mitochondrial Ca^2+^ uptake through the MCU is detrimental to mitochondrial function and can cause down‐regulation of mitochondrial membrane potential (MMP), up‐regulation of reactive oxygen species (ROS) and suppression of ATP production.^7^ Cellular and mitochondrial accumulation of Ca^2+^ leads to the activation of mitochondrial permeability transition pore. Subsequently, the pro‐apoptotic proteins, such as cytochrome c, are released from mitochondrion to cytoplasm, resulting in apoptosis.^8^, ^9^

Iron overload is a crucial factor involved in mitochondrial dysfunction in brain. Accumulation of iron concentration has been observed in many neurological diseases, such as TBI^10^, ^11^ and intracerebral haemorrhage.^12^ In neurons, iron exists in transferrin (Tf)‐iron form, which is regulated by transferrin receptor protein (TfR). When neurons are injured, the Tf‐iron‐TfR complex is dissociated, and subsequently iron is released to cytoplasm.^13^ Iron accumulation in cytoplasm may lead to cell death by destroying mitochondrial function, resulting in ROS generation and oxidative stress.^14^ Recently, a growing number of researches have demonstrated that MCU was related to iron overload‐triggered dysfunction of mitochondrion. It has been proposed that RU360, a MCU inhibitor, prevented iron overload‐caused mitochondrial dysfunction in brain via attenuating production of ROS and depolarization of mitochondrion.^15^ In addition, blockage of MCU could decrease the accumulation of iron in cytoplasm, alleviate apoptosis and brain injury following SAH by improving cell energy supply and suppressing ROS generation.^16^

Overall, MCU is a major pathway for mitochondrial Ca^2+^ and iron uptake. However, the accurate role of MCU‐mediated Ca^2+^ and iron accumulation in TBI is unclear. The present study is aimed to illustrate the accumulation of Ca^2+^ and iron after TBI and to investigate the potential role of MCU as a therapeutic target in TBI.

## 2 MATERIALS AND METHODS

### 2.1 Animals

This study was carried out in accordance with the recommendations of guide for the Care and Use of Laboratory Animals by National Institutes of Health (NIH). The protocol was approved by the Animal Care and Use Committee of Nanjing University. Male ICR mice (28‐32 g) were obtained from Animal Center of Jinling hospital (Nanjing, China). Mice were housed on a 12 h light/dark cycle at 23 ± 1°C with free access to food and water.

### 2.2 Primary culture of mouse cortical neurons

The culture of mouse cortical neurons was performed according to previous studies.^17^, ^18^ In brief, mouse cortical neurons were isolated from the embryos of time‐mated pregnant mice and subsequently cultured in poly‐D‐lysine‐coated six‐well dishes at a density of 1 × 10^6^ cells per well. Then, the neurons were cultured in neurobasal medium (Life Technologies, USA, catalogue number: 21103049) supplemented with 2% B27 (Life Technologies, USA, catalogue number: 17504044) and 1 mmol/L glutamate (Sigma Aldrich, USA, catalogue number: G3291) at 37°C on 5% CO_2_ incubator. Half of the culture medium was replaced with fresh medium every 3 days. The in vitro studies were performed after culture of 10‐12 days.

### 2.3 Models of TBI

The in vivo (mouse) TBI model was performed with a modified version of the weight‐drop model.^19^ As described in our previous study,^20^ mice were anaesthetized with an intraperitoneal (ip) injection of chloral hydrate (1%, 5 ml/kg) and subsequently placed on the platform under the weight of the weight‐drop device. With a 1.5 cm midline longitudinal incision of the scalp, the skull was exposed. Upon the impact area was situated at the left anterior frontal region (1.5 mm lateral to the midline on the mid‐coronal plane), the weight was released from the height of 2.5 cm and dropped along a stainless steel string on the skull. The mortality results from apnoea were decreased by early respiratory support. Then, the mice were returned to cages to recover for 24 hours with free access to a standard diet. The sham‐injured mice underwent the same procedures, but did not undergo the weight drop.

The in vitro (neuron) model of TBI was conducted according to previous studies.^17^, ^18^ Briefly, the six‐well plates were manually scratched with a sterile plastic needle followed by a 9 × 9 square grid (the space between every line was 4 mm). Then, neurons were cultured at 37°C in 5% CO_2_ incubator for another 24 hours without change of culture medium.

### 2.4 Groups and drug administration

Both RR (catalogue number: R2751) and Sper (catalogue number: 85590) were purchased from Sigma‐Aldrich. For in vivo studies, mice were divided into nine groups: sham, TBI, TBI + vehicle, TBI + RR (1 mg/kg, 3 mg/kg, 5 mg/kg) and TBI + Sper (2 mg/kg, 5 mg/kg, 10 mg/kg). RR and Sper were dissolved in sterile saline solution and given by ip administration 30 min after TBI. The doses of RR (1 mg/kg, 3 mg/kg, 5 mg/kg) and Sper (2 mg/kg, 5 mg/kg, 10 mg/kg) used in our study were based on previous reports^21^, ^22^ and our preliminary experiments.

For in vitro studies, neuronal cells were divided into five groups: control, TBI, TBI + vehicle, TBI + RR and TBI + Sper. RR and Sper were firstly dissolved in dimethyl sulfoxide and then added to medium to achieve different final concentrations. The concentrations of RR (10 μmol/L) and Sper (10 μmol/L) used was based on previous studies^23^, ^24^ and our preliminary experiments.

### 2.5 Neurological deficit and brain water content

The neurologic status of mice was estimated in 1, 3 and 7 days following TBI by the neurological severity score (NSS) and grip test.^26^ For NSS, the investigators evaluate the ability of mouse to perform 10 different tasks that indicate balance, motor function and alertness. One point is given for failing to perform each task, therefore 10 = maximum deficit and 0 = minimum deficit (Table 1). For grip test, mice were placed onto a 45 cm long wire. The score criterion of grip test was shown in Table 2. Both tests were performed in tree times and a total point was calculated for each mouse. Both the neurobehavioral tests were carried out by two investigators who were blinded to the experimental groups.

**Table 1:** Neurological severity scoring

Items	Description	Points	
Exit circle	Ability and initiative to exit a circle of 30 cm diameter (time limit: 3 min)	Success	Failure
Mono‐/hemiparesis	Paresis of upper and/or lower limb of contralateral side	0	1
Straight walk	Alertness, initiative and motor ability to walk straight, when placed on the floor	0	1
Startle reflex	Innate reflex (flinching in response to a loud hand clap)	0	1
Seeking behaviour	Physiological behaviour as a sign of "interest" in the environment	0	1
Beam balancing	Ability to balance on a beam 7 mm in width for at least 10 s	0	1
Round stick balancing	Ability to balance on a round stick 5 mm in diameter for at least 10 s	0	1
Beam walk: 3 cm	Ability to cross a beam(length ×width, 30 × 3 cm)	0	1
Beam walk: 2 cm	Same task but with increased difficulty (beam width=2 cm)	0	1
Beam walk: 1 cm	Same task but with increased difficulty (beam width=1 cm)	0	1
Maximum score			10

**Table 2:** Grip test scoring

Description	Points
Mouse was unable to remain on the wire for less than 30 s	0
Mouse failed to hold on to the wire with both forepaws and hind paws together	1
Mice held on to the wire with both forepaws and hind paws but not the tail	2
Mouse used its tail along with both forepaws and both hind paws	3
Mouse moved along the wire on all four paws plus tail	4
Mice that scored four points also ambulated down one of the posts used to support the wire	5

The brain water content was conducted depend on a previous study.^27^ Briefly, mouse brain was taken out and placed onto a cooled brain matrix 1 day following TBI. The cerebellum and stem were taken away and the ipsilateral tissue was weighed to get the wet weight (ww). Then, the hemisphere was dried for 72 hours at 80°C and weighed again to get the dry weight (dw). The brain water content equals (ww − dw)/ww × 100%.

### 2.6 Measurement of lesion volume

The measurement of lesion volume of the brain was conducted according to a previous study.18 Briefly, mice were killed 7 days after TBI. The brains were collected, fixed for 12 hours and cryoprotected in 15% sucrose in PBS. The brain sections (500 mm intervals spanning the length of the brain) were stained with cresyl violet. The areas of the lesion, injured as well as non‐injured cortex and hemisphere were estimated using an image analysis system. Area measurements from each section were obtained and summed, and corresponding volumes were calculated. Lesion volume was quantitatively analysed with Image‐Pro Plus system and was showed as percent (the percentage volume of the non‐injured hemisphere).

### 2.7 Isolation of mitochondria

Tissue mitochondria were isolated by differential centrifugation, using a Functional Mitochondria Isolation Kit (Genmed Scientifics Inc, USA, catalogue number: GMS10006). The freshly removed brain tissue was weighed and rapidly placed in ice‐cold washing liquid (provided with the kit, Reagent A) to dislodge impurities. It was then crumbled and homogenized in Glass/Teflon Potter homogenizer with isolating fluid (prepped prior using the provided physic liquor in the kit, Reagent F: Reagent C: Reagent B = 1:50:200), then the homogenate was centrifuged at 1500 g for 10 minutes and the supernatant was retained (the nucleus and unsolvable cells were removed). The supernatant was centrifuged at 10 000 g for 10 minutes and the precipitates were saved (mitochondrial pellets were isolated). The isolated mitochondria were conserved in preservative fluid (Reagent E in the kit) and either stored at −80°C or used.

### 2.8 Measurement of mitochondrial and cellular Ca^2+^ concentration

The mitochondrial and cellular Ca^2+^ concentrations were measured by Fura‐3‐AM with a mitochondrial Ca^2+^ concentration quantitative determination kit (Genmed Scientifics Inc, USA, catalogue number: GMS10153) and a cellular Ca^2+^ concentration quantitative determination kit (Genmed Scientifics Inc, USA, catalogue number: GMS10152). The isolated mitochondria and cytoplasm were washed three times in Hanks’ solution and then Fura‐3‐AM (5 μmol/L) was added in. The samples with Fura‐3‐AM were incubated in 37°C for 30 minutes. Then, the samples were washed with Hanks’ solution three times to dislodge the excess Fura‐3‐AM and incubated at 37°C for 30 minutes again. The fluorescence intensity was determined at an excitation of 506 nm and emission of 526 nm using a fluorescence microplate reader.

### 2.9 Measurement of cellular ROS and ATP levels

The levels of ROS were measured by a fluorescence assay kit (Genmed Scientifics Inc, USA, catalogue number: GMS10016.3) and the levels of ATP were analysed by a ATP assay kit (Beyotime, China, catalogue number: S0026) based on the instruction from the manufacturer.

### 2.10 Western blot analysis

Western blot analysis was performed according to previous studies.^28^ Proteins were separated by 8%‐12% sodium dodecyl sulphate‐polyacrylamide gel electrophoresis, transferred to polyvinylidene fluoride membranes and incubated with primary antibodies at 4°C overnight. The primary antibodies used were dynamin‐related protein1 (Drp1) (1:1000; Abcam, USA, catalogue number: ab56788), mitochondrial elongation factor 1 (MIEF1) (1:1000; Abcam, USA, catalogue number: ab89944), Fis1 (1:1000; Abcam, USA, catalogue number: ab71498), TfR (1:1000; Abcam, USA, catalogue number: ab84036), ferritin (Ft) (1:1000; Abcam, USA, catalogue number: ab75973), ferroportin‐1 (Fpn‐1) (1:1000; Abcam, USA, catalogue number: ab58695), iron regulatory protein‐1 (IRP‐1) (1:1000; Cell Signaling Technology, USA, catalogue number: 20272), iron regulatory protein‐2 (IRP‐2) (1:1000; Cell Signaling Technology, USA, catalogue number: 37135), cytochrome c (1:5000; Abcam, USA, catalogue number: ab133504), COX IV (1:1000; Cell Signaling Technology, USA, catalogue number: 11967), caspase‐3 (1:1000; Cell Signaling Technology, USA, catalogue number: 9661) and β‐actin (1:5000; Bioworld Technology, USA, catalogue number: AP0060). Then, the membranes were incubated with corresponding secondary antibodies at room temperature for 2 hours.

### 2.11 Iron staining

For iron staining, the 7 μm frozen sections were washed with phosphate buffer solution (PBS) and incubated with fresh‐made Prussian blue staining solution (2% HCl and 2% K_4_[Fe(CN)_6_]) in the dark for 60 minutes. After washing with PBS again, the sections were incubated with fresh‐made diaminobenzidine solution (1 mL 3% H_2_O_2_, 40 mL 1 mol/L Tris pH 7.5 and 30 mg DAB tablet) in the dark. Finally, the sections were mounted in glycerinum. Six pictures (400×) from each section were taken and quantitation was performed with Image‐Pro Plus system.

### 2.12 Transmission electron microscope (TEM) observation

We used TEM to identify mitochondria as previously described.^29^ Briefly, at 1 day after TBI, the mice were killed and perfused with 2.5% buffered glutaraldehyde. The specimens were collected and fixed in glutaraldehyde with a 1% (w/v) solution of osmium tetroxide. The fixed specimens were then embedded, sectioned, double stained with lead citrate and uranyl acetate, observed under a TEM JEM‐1011 (JEOL, Japan).

### 2.13 Nissl staining

For Nissl staining, the sections (4 μm) were hydrated with 1% toluidine blue for 20 min at 50°C. After washing with double distilled water, the sections were dehydrated and mounted with permount. For quantification, six random high‐power fields (400×) surrounding contusion were chosen and the mean number of surviving neurons in the six fields was considered as the data of each section. A total of four sections from each animal were used for quantification. The final average number of the four sections was regarded as the data for each sample. Data were presented as the number of neurons per high‐power field. All the processes were performed by two independent pathologists who had no prior knowledge of the group assignments.

### 2.14 TUNEL staining

The TUNEL staining was conducted using an In Situ Cell Death Detection Kit (Roche Inc, USA, catalogue number: 11684817910) based on our previous studies.^18^ In brief, the sections were incubated with labelling solution containing TUNEL at 37°C for 1 hour. The brain injury was expressed by the apoptotic index, known as the average percentage of apoptotic cells in each section counted in six cortical microscopic fields (400×). The apoptotic cells were measured by two independent observers blinded to the groups.

### 2.15 Cell viability analysis

To analyse the cell viability of primary cultured neuron, we first used the trypan blue (TB) staining assay. Cells were stained by 0.4% TB (Beyotime Biotech Inc, China, catalogue number: ST798) after treatment. Stained cells were considered as dead while unstained cells were considered as viable. The number of TB‐positive cells and total cell number were counted. Survival value = (number of stained cells/number of total cells) × 100%.

In addition, a lactate dehydrogenase (LDH) cytotoxicity assay kit (Beyotime Biotech Inc, China, catalogue number: C0016) was used to confirm the results of TB staining. In brief, cells were treated with LDH release agent and the culture medium was centrifuged. The supernatant was further collected to evaluate the activity of LDH. The OD value at 490 nm was analysed by a spectrophotometer. The percentage of damaged cells (%) = (OD490_sample_ – OD490_media_)/(OD490_maximum_ – OD490_media_) × 100%. OD490_maximum_ = cells treated with LDH release agent and OD490_media_ = only media without any cells.

### 2.16 Measurement of neuron ROS levels

The levels of ROS in neuron were measured with a ROS assay kit (Beyotime Biotech Inc, China, catalogue number: S0033). Briefly, cells were stained with 2′,7′‐dichlorodihydrofluorescein diacetate (DCF‐DA, 10 mmol/L) at 37°C for 10 minutes. Then, cells were washed with PBS three times and observed immediately under a fluorescent microscopy (Carl Ziess, Germany). The fluorescence images were collected using a single rapid scan. In addition, we analysed the levels of ROS by a fluorescence microplate reader (BioTek USA). Cells were stained with DCFH‐DA, rinsed with PBS and centrifuged for 5 minutes at 1000 *g*, the supernatant was removed and the remaining cells were resolved with 1% Triton X‐100. Fluorescence was analysed at an excitation wavelength of 488 nm and at an emission wavelength of 525 nm.

### 2.17 Statistical analysis

All statistical analyses were performed with SPSS 19.0 (SPSS Inc, Chicago, IL). Each experiment was repeated at least three times. Continuous variables were presented as mean ± SEM. For the behavioural tests, Kruskal‐Wallis and Mann‐Whitney *U* analysis were used to compare the data between multiple experimental groups because they were categorical variables. For other assays, one‐way analysis of variance (ANOVA) followed by Tukey's test was used. A value of *P* < 0.05 was considered statistically significant.

## 3 RESULTS

### 3.1 General observations and the mortality rate of mice

A total of 398 mice were used in this study, among them 51 mice died during the operation. The mortality of mice within 24 hours in each group was as follows: sham group 0% (0 of 55 mice), TBI group 15.4% (10 of 65 mice), TBI + vehicle group 12.7% (8 of 63 mice), TBI + 1 mg/kg RR group 14.3% (3 of 21 mice), TBI + 3 mg/kg RR group 15.4% (10 of 65 mice), TBI + 5 mg/kg RR group 18.2% (4 of 22 mice), TBI + 2 mg/kg Sper group 14.3% (3 of 21 mice), TBI + 5 mg/kg Sper group 14.1% (9 of 64 mice), TBI + 10 mg/kg Sper group 18.2% (4 of 22 mice). There were no significant differences in mortality among the TBI, TBI + vehicle, TBI + RR and TBI + Sper groups (data not shown).

### 3.2 RR and Sper provided neuroprotection after TBI

**Figure 1:**
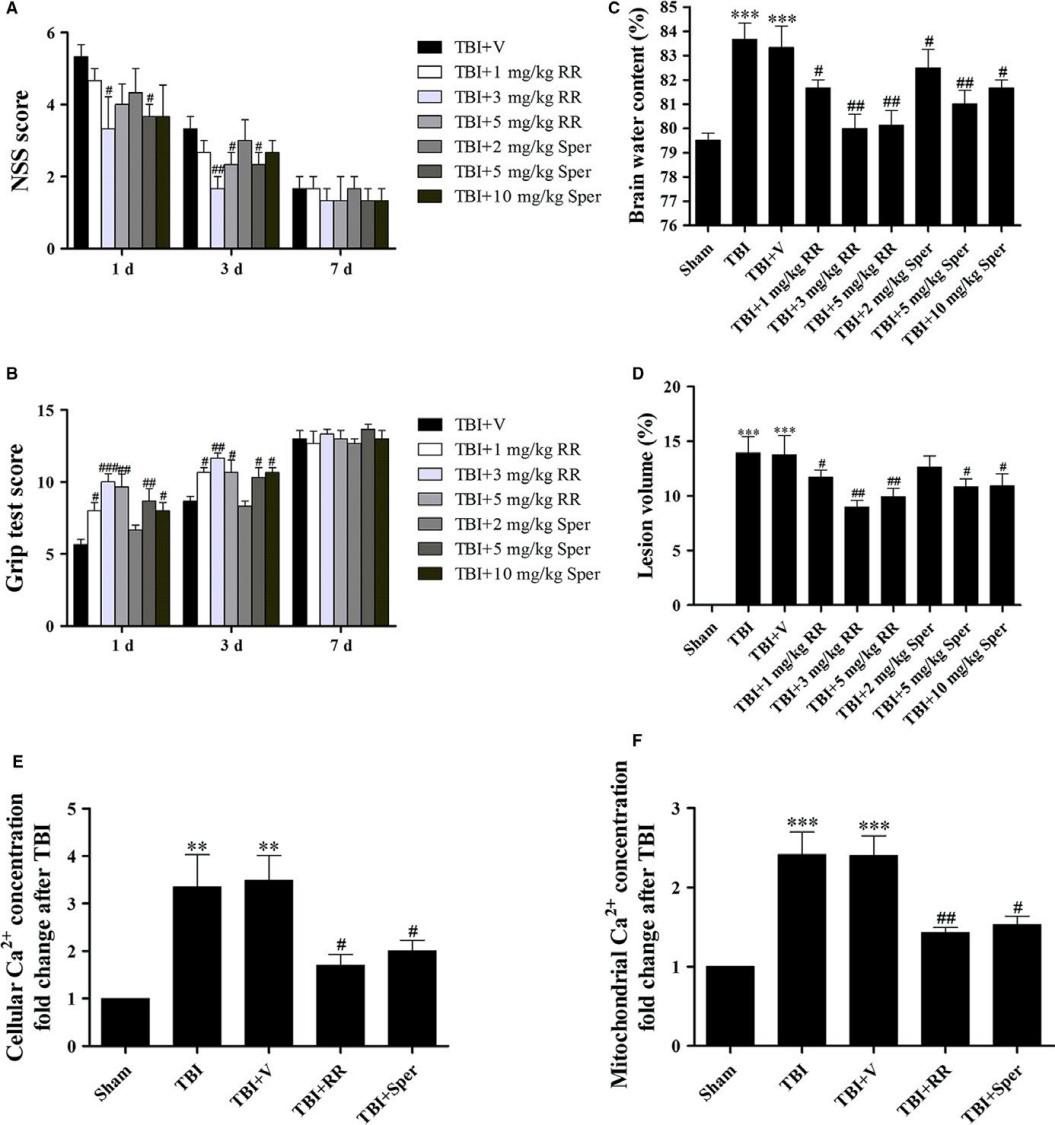
Administration of ruthenium red (RR) or Sper protected mice against secondary brain injury and decreased Ca^2+^ concentrations after traumatic brain injury (TBI). (A, B, C) Mice were subjected to TBI and then received 1 mg/kg, 3 mg/kg, 5 mg/kg of RR or 2 mg/kg, 5 mg/kg, 10 mg/kg of Sper ip injection or vehicle 30 min after TBI. NSS and Grip test score were evaluated at 1, 3 and 7 days after TBI while brain water content was examined at 1 day after TBI. (A, B) All doses of RR or Sper had an improved motor performance within 3 days; however, larger doses such as 5 mg/kg of RR and 10 mg/kg of Sper did not exhibit a better neuroprotection. This effect was no longer significant at 7 days after TBI, n = 6 per group. (C) Mice subjected to TBI or treated with vehicle had an increased brain water content as compared with the sham group. Brain water content was significantly lower in the groups treated with RR or Sper than the vehicle‐treated group. Moreover, doses of 3 mg/kg of RR and 5 mg/kg of Sper had the best effect in relieving brain oedema, n = 6 each group. (D) TBI‐induced profound tissue loss of the brain was reversed by RR or Sper, and doses of 3 mg/kg of RR and 5 mg/kg of Sper had the best effect. (E, F) RR or Sper treatment decreased Ca^2+^ concentration following TBI. Restored cellular (E) and mitochondrial (F) concentrations of Ca^2+^ by RR (3 mg/kg) or Sper (5 mg/kg) treatment after TBI, n = 6 each group. Data are presented as mean ± SEM; **P < 0.01, ***P < 0.001 vs sham group; ^#^ *P* < 0.05, ^##^ *P* < 0.01, ^###^ *P* < 0.001 vs TBI + vehicle group. Scale bar: 50 μm

To determine whether regulation of MCU could provide neuroprotective effects following TBI, we set nine groups as follows: sham, TBI, TBI + vehicle, TBI + RR (1 mg/kg, 3 mg/kg, 5 mg/kg) and TBI + Sper (2 mg/kg, 5 mg/kg, 10 mg/kg). Firstly, we used NSS and grip test to study the motor performance of mice after TBI. Our results indicated that the RR‐treated mice showed better motor performance than that of the vehicle‐treated mice at 1 day (Figure 1A, B). In addition, at 3 days, a significant difference was still detectable. However, there was no significant difference between these two groups at 7 days (*P* > 0.05). Surprisingly, the mice treated with Sper also presented better motor performance than the TBI + vehicle group (Figure 1A,B).

We further performed brain water content to confirm the neuroprotection of RR and Sper. Compared with the sham group, brain water content was significantly increased at 1 day after TBI (Figure 1C). However, it was remarkably decreased by treatment of RR. Surprisingly and consistently, the brain water content in TBI mice treated with Sper was also decreased compared to the TBI + vehicle group (Figure 1C). Collectively, our results suggested that both RR and Sper were neuroprotective against TBI. Importantly, both experiments above suggested that larger doses such as 5 mg/kg of RR and 10 mg/kg of Sper did not provide a better neuroprotection (Figure 1A‐C).

Then we determined whether RR or Sper treatment may affect TBI‐caused cortical lesion volume. Figure 1D showed that TBI induced obvious brain tissue loss. However, both RR and Sper decreased TBI‐induced lesion volume (Figure 1D). Combined with the effects of Sper in NSS, grip test and brain water content, our result suggested that Sper could provide neuroprotection after TBI probably by inhibiting MCU, and suggested that doses of 3 mg/kg of RR and 5 mg/kg of Sper showed the best effects, which we used in our subsequent experiments (Figure 1A‐D).

### 3.3 RR and Sper decreased TBI‐induced up‐regulation of cellular and mitochondrial Ca^2+^ concentrations

Calcium homeostasis is necessary for physiological cell functions and the destruction of Ca^2+^ homeostasis may aggravate secondary brain injury following TBI. In our study, both cellular and mitochondrial Ca^2+^ concentrations were measured. The data demonstrated that the Ca^2+^ concentrations in both cytoplasm and mitochondrion were increased in TBI and TBI + vehicle groups. Nevertheless, treatment with RR or Sper significantly decreased cellular and mitochondrial Ca^2+^ concentrations (Figure 1E‐F). This result suggested that both RR and Sper could inhibit MCU or prevent Ca^2+^ accumulation to protect mice from TBI.

### 3.4 RR and Sper reversed TBI‐induced cellular iron accumulation

**Figure 2:**
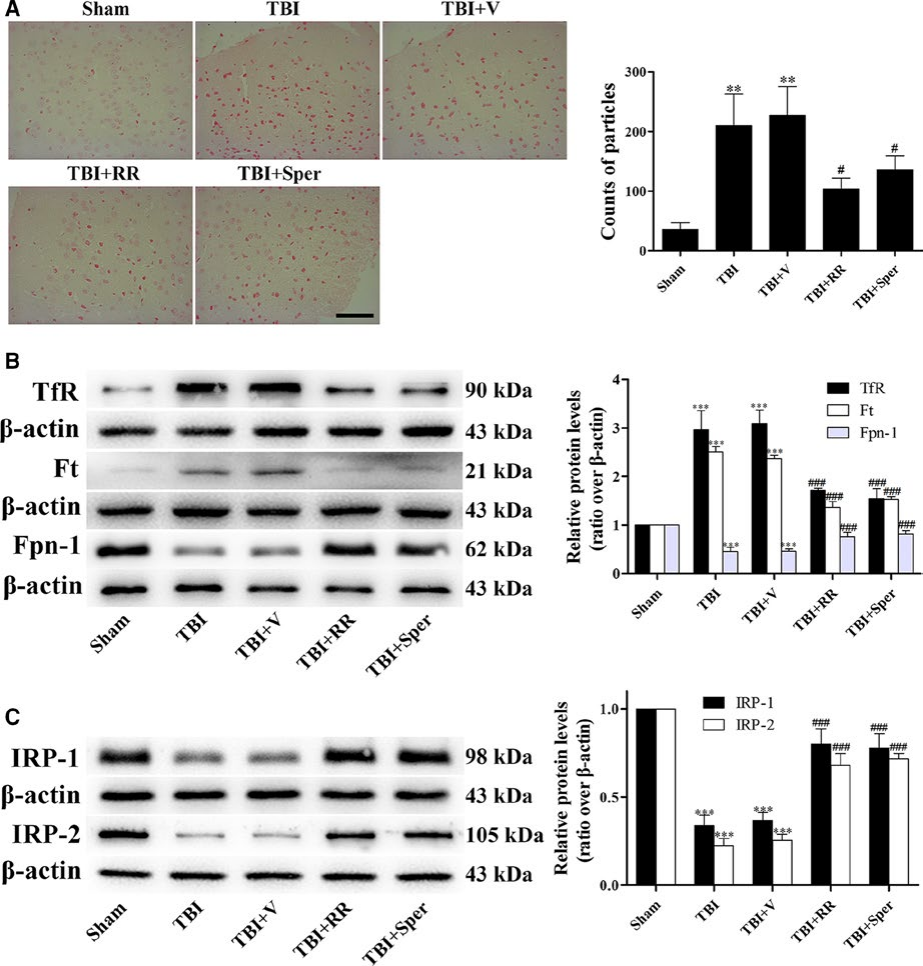
TBI induced cellular iron accumulation while ruthenium red (RR) or Sper administration partially reversed the effects. (A) Mice were subjected to traumatic brain injury (TBI) and then administrated RR (3 mg/kg), Sper (5 mg/kg) or vehicle 30 min after TBI. Ipsilateral cortex was collected 1 day after TBI and then subjected to iron staining. The left panel shows representative brain sections of cortex with iron stained (red) cells. The right panel is quantification of the digitized images showing the count of particles and fraction of total iron staining in each group. (B, C) TBI‐induced disruption of cellular iron homeostasis was improved by RR or Sper. Mice were subjected to TBI and treated with RR (3 mg/kg), Sper (5 mg/kg) or vehicle 30 min after TBI. Ipsilateral brain tissues were collected 1 day after TBI. The protein levels of TfR, Ft, Fpn‐1, IRP‐1 and IRP‐2 were measured by Western blot. TfR and Ft proteins were up‐regulated while Fpn‐1, IRP‐1 and IRP‐2 proteins were down‐regulated after TBI, however RR or Sper treatment reversed these effects. Data are presented as mean ± SEM, n = 6 per group; **P < 0.01, ***P < 0.001 vs sham group; ^#^ *P* < 0.05, ^###^ *P* < 0.001 vs TBI + vehicle group. β‐actin was used as a loading control

Iron homeostasis is closely mediated by Tf‐TfR internalization for iron uptake, Ft for iron storage and Fpn‐1 for iron export in cells. In our study, cellular iron was accumulated after TBI as proven by iron staining (Figure 2A). Moreover, the expression of TfR and Ft was obviously increased and the expression of Fpn‐1 was decreased (Figure 2B), suggesting that iron was transported to cells. However, treatment with RR or Sper significantly attenuated the accumulation of iron (Figure 2A), down‐regulated the protein levels of TfR and Ft, and up‐regulated the protein levels of Fpn‐1 compared to the TBI + vehicle group (Figure 2B), demonstrating that TBI‐induced accumulation of iron was reversed by inhibition of MCU.

### 3.5 RR and Sper prevented TBI‐triggered disruption of cellular iron homeostasis

The iron‐related proteins such as TfR, Fpn‐1 and Ft could be modulated by IRP‐1 and IRP‐2. To further confirm the accumulation of iron in brain cells, we examined the expression of IRP‐1 and IRP‐2 by Western blot. Our data indicated that the expression of IRP‐1 and IRP‐2 significantly decreased after TBI. Treatment with RR or Sper obviously reversed the expression of IRP‐1 and IRP‐2 (Figure 2C), suggesting that TBI‐triggered disruption of iron homeostasis was restored via inhibition of MCU.

### 3.6 TBI elevated ROS Levels and limited mitochondrial ATP generation

**Figure 3:**
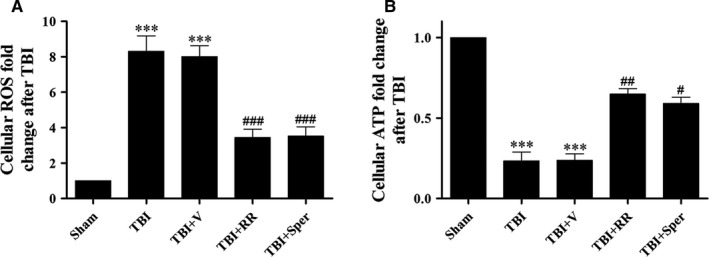
Treatment of ruthenium red (RR) or Sper stabilized ROS and ATP homeostasis following traumatic brain injury (TBI). (A) Cellular ROS levels after TBI were repressed by RR (3 mg/kg) or Sper (5 mg/kg) administration. (B) Cellular ATP was resupplied after TBI by RR (3 mg/kg) or Sper (5 mg/kg) administration. Data are presented as mean ± SEM, n = 6 per group; ***P < 0.001 vs sham group; ^#^ *P* < 0.05, ^##^ *P* < 0.01, ^###^ *P* < 0.001 vs TBI + vehicle group

Calcium and iron overload may lead to excessive generation of ROS and insufficient supply of ATP. Therefore, to investigate whether ROS production and ATP reduction after TBI was associated with mitochondrial Ca^2+^ and iron overload, we measured ROS and ATP levels. In the sham group, low levels of ROS were observed. However, a significant increase in ROS generation was observed in the TBI and TBI + vehicle groups. After treatment with RR or Sper, the levels of ROS were evidently less than the TBI + vehicle group (Figure 3A). In addition, compared to the sham group, the ATP levels in TBI and TBI + vehicle groups decreased dramatically. However, RR or Sper treatment fundamentally increased the ATP supply (Figure 3B).

### 3.7 RR and Sper ameliorated TBI‐induced deformation of mitochondria

**Figure 4:**
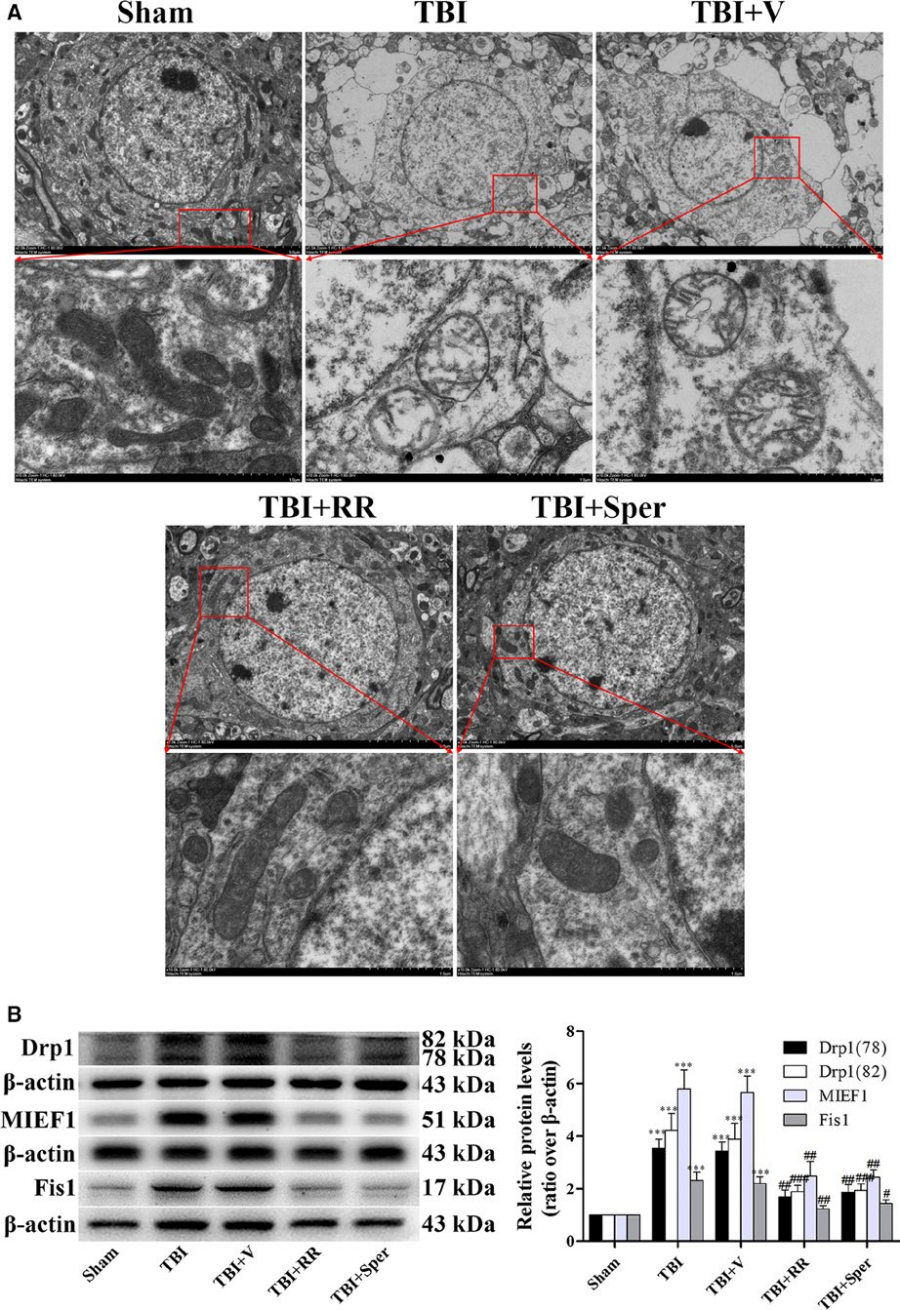
Treatment of ruthenium red (RR) or Sper ameliorated traumatic brain injury (TBI)‐induced deformation of mitochondria. (A) Electron photomicrographs of mitochondria after TBI. A representative of a mitochondrion with normal shape from the cortex of the sham group, a swelling mitochondrion with collapsed cristae from the cortex of the TBI group, a mitochondrion with quite normal shape and clear cristae of the TBI + RR (3 mg/kg) and TBI + Sper (5 mg/kg) groups. (B) Comparison of the expression of Drp1, MIEF1 and Fis1 using Western blot. Mice were subjected to TBI and treated with RR (3 mg/kg), Sper (5 mg/kg) or vehicle 30 min after TBI. Ipsilateral brain tissues were collected 1 day after TBI and the protein levels of Drp1, MIEF1 and Fis1 were measured by Western blot. TBI increased the protein levels of Drp1, MIEF1 and Fis1, while RR or Sper treatment significantly decreased them. Data are presented as mean ± SEM, n = 6 per group; ***P < 0.001 vs sham group; ^#^ *P* < 0.05, ^##^ *P* < 0.01, ^###^ *P* < 0.001 vs TBI + vehicle group. β‐actin was used as a loading control for cytoplasmic and whole‐cell extracts

Mitochondrial Ca^2+^ and iron accumulation, as well as increased ROS generation and insufficient ATP supply, may lead to mitochondria swelling. In order to evaluate the mitochondrial injury following TBI, we used TEM to observe the mitochondrial morphology. In the sham group, TEM showed intact mitochondria, with double membranes visible, cristae structurally intact and round in shape. Conversely, the mitochondrial morphology of TBI and TBI + vehicle groups were swelling shape, near disappearance of the cristae and disruption of the membranes, which lost typical mitochondrial structure (Figure 4A). However, RR or Sper treatment protected the mitochondrion from deformation (Figure 4A).

### 3.8 The expression of protein related to mitochondrial morphology

To explain the mechanisms of mitochondrial morphology changes after TBI, we examined some proteins that are associated with mitochondria fission and fusion. Drp1 and Fis1 are key proteins regulating mitochondrial fission, and MIEF1 is involved in mitochondria fusion. Therefore, we examined the expression of these proteins after TBI. Compared to sham group, the expression of Drp1, Fis1 and MIEF1 increased significantly in TBI and TBI + vehicle groups (Figure 4B). However, when TBI mice was treated with RR or Sper, the expression of these proteins was apparently decreased (Figure 4B). These results indicated that blockage of MCU reversed TBI‐induced change of Drp1, Fis1 and MIEF1, thus protecting the mitochondria from deformation.

### 3.9 RR and Sper reversed TBI‐induced neuron death

**Figure 5:**
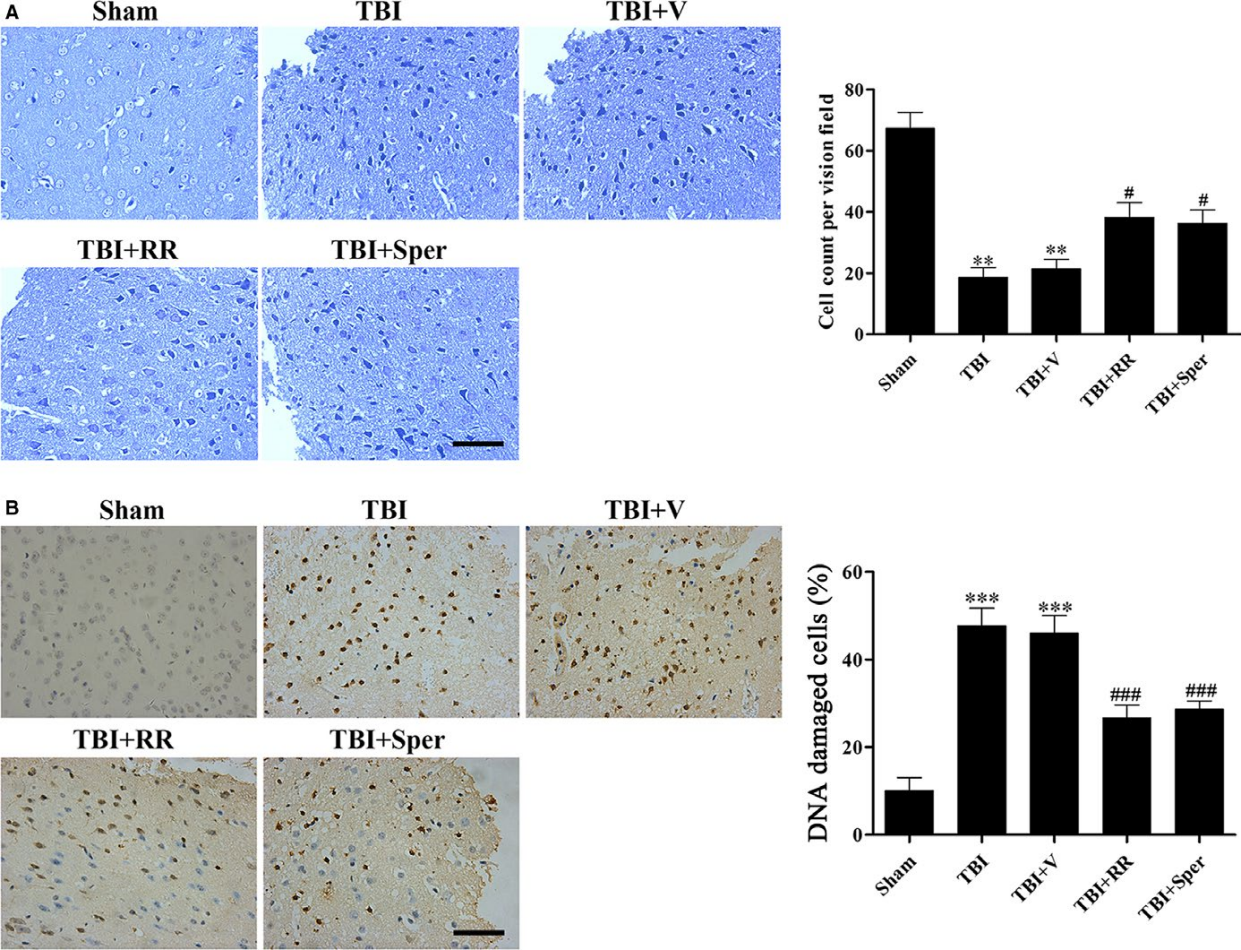
Ruthenium red (RR) or Sper treatment suppressed traumatic brain injury (TBI)‐induced neuronal cell death and DNA damage. (A) Nissl staining to visualize the neuronal cell outline and structure. TBI reduced the number of the neurons and treatment of RR (3 mg/kg) or Sper (5 mg/kg) preserved neurons from damage. (B) DNA damage was determined using TUNEL assays 1 day after TBI. The DNA damaged cells were significantly increased after TBI compared to the sham group. RR (3 mg/kg) or Sper (5 mg/kg) treatment significantly decreased the percentage of DNA damaged cells after TBI. Data are presented as mean ± SEM, n = 6 each group; **P < 0.01, ***P < 0.001 vs sham group; ^#^ *P* < 0.05, ^###^ *P* < 0.001 vs TBI + vehicle group. Scale bar: 50 μm

The damage of mitochondrion following TBI could cause the release of pro‐apoptotic proteins from mitochondrion to cytosol, leading to apoptosis. Moreover, TBI‐induced apoptosis in the early response may result in neuron death. To verify the occurrence of neuron death, we applied Nissl staining to clarify the morphology change and number of cortex neurons after TBI. In the sham group, the neurons were intact and clear, without oedema around the cells. While in the TBI and TBI + vehicle groups, the damaged neuron cells was increased, exhibiting extensive degenerative changes including swollen cell bodies, shrunken cytoplasma, sparse cell arrangements and oval or triangular nucleus. On the contrary, the severity of neuron damage in the TBI + RR or TBI + Sper groups was significantly alleviated (Figure 5A).

### 3.10 RR and Sper blocked TBI‐induced DNA damage and apoptosis

To examine TBI‐induced DNA damage of neural cells, we used TUNEL staining. Results showed that in the sham group, few DNA damaged cells were detected. However, a growing number of cells with chromatin condensation and fragmented nuclei were found in the TBI and TBI + vehicle groups. RR or Sper administration remarkably decreased the number of DNA damaged cells (Figure 5B).

**Figure 6:**
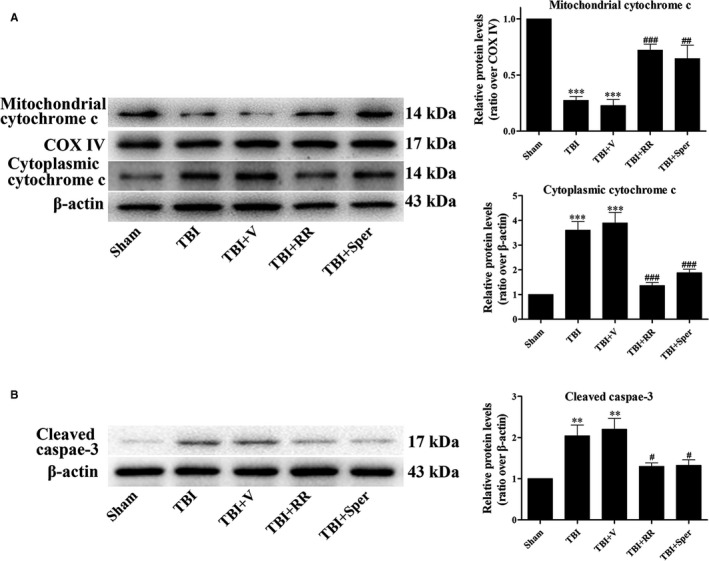
Ruthenium red (RR) or Sper treatment inhibited traumatic brain injury (TBI)‐induced up‐regulation of apoptosis‐related proteins. (A, B) Mice were subjected to TBI and treated with RR (3 mg/kg), Sper (5 mg/kg) or vehicle 30 min after TBI. Ipsilateral brain tissues were collected 1 day after TBI and the protein levels of cytochrome c and cleaved caspase‐3 were measured by Western blot. RR or Sper treatment significantly decreased apoptosis induced by TBI. Data are presented as mean ± SEM, n = 6 per group; **P < 0.01, ***P < 0.001 vs sham group; ^#^ *P* < 0.05, ^##^ *P* < 0.01, ^###^ *P* < 0.001 vs TBI + vehicle group. COX IV was used as a loading control for mitochondria extracts. β‐actin was used as a loading control for cytoplasmic and whole‐cell extracts

To further examine the effects of RR and Sper on apoptosis induced by TBI, we analysed several apoptosis markers such as cytochrome c and caspase‐3. Our data showed that the mitochondrial cytochrome c was decreased while the cytoplasmic cytochrome c and cleaved caspase‐3 were increased after TBI (Figure 6A,B). However, treatment of RR or Sper evidently reduced the TBI‐induced apoptosis compared to the vehicle‐treated group (Figure 6A,B).

### 3.11 RR protected primary cultured neurons from TBI

**Figure 7:**
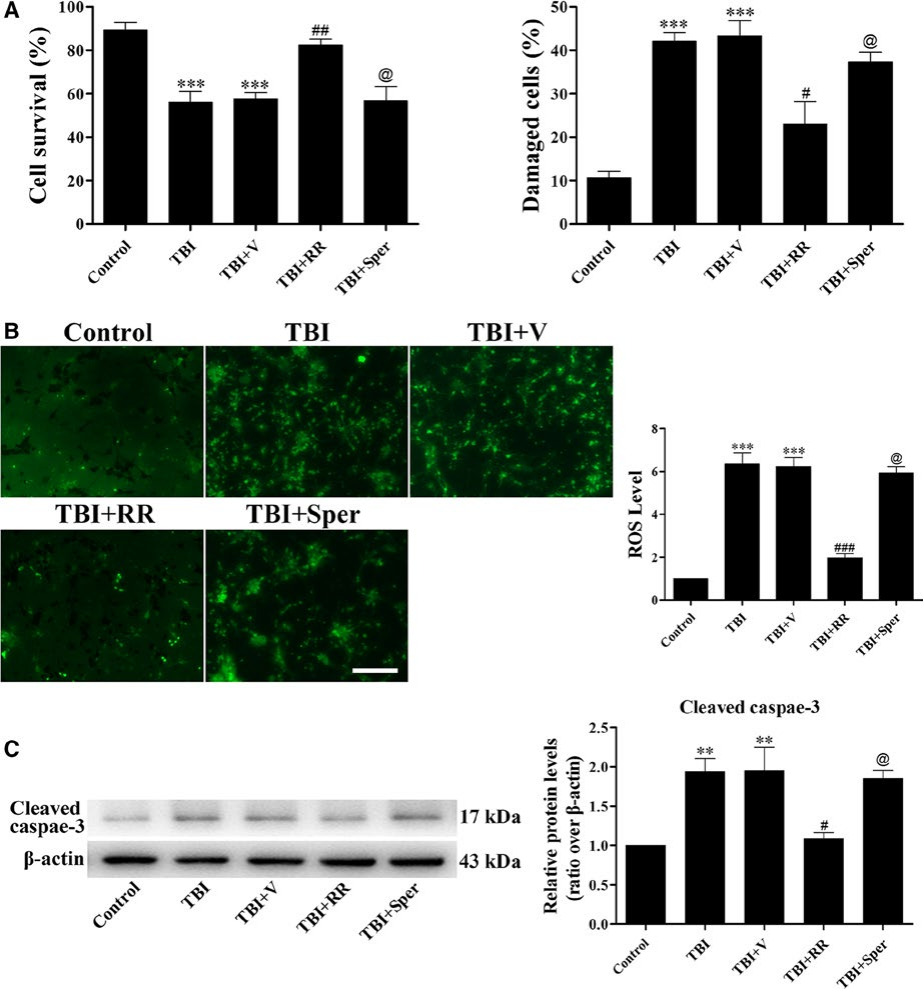
Ruthenium red (RR) instead of Sper treatment protected primary cultured neurons from traumatic brain injury (TBI). (A) Primary cortical neurons were subjected to scratch injury and then treated with RR (10 μM), Sper (10 μM) or saline for 1 day. The LDH release assay and TB staining were used to evaluate cell viability. The percentage of damaged cells significantly increased after TBI compared to the control group. RR treatment significantly decreased damaged cells after TBI, however, Sper treatment had no significant impact compared to the TBI + vehicle group. (B) Cells were subjected to scratch injury and subsequently treated with RR (10 μM), Sper (10 μM) or saline for 1 day. Then cells were incubated with DCFH‐DA and subjected to fluorescent microscopy analysis. The intracellular ROS was significantly increased after TBI compared to the sham group, and administration of RR instead of Sper significantly repressed ROS production as compared to the TBI + vehicle group. (C) RR (10 μM) treatment significantly decreased the expression of cleaved caspase‐3 after TBI, however, Sper treatment had no obvious effect. Data are presented as mean ± SEM, n = 6 per group; **P < 0.01, ***P < 0.001 vs control group; ^#^ *P* < 0.05, ^##^ *P* < 0.01, ^###^ *P* < 0.001 vs TBI + vehicle group; ^@^ *P* > 0.05 vs TBI + vehicle group. β‐actin was used as a loading control. Scale bar: 50 μm

**Figure 8:**
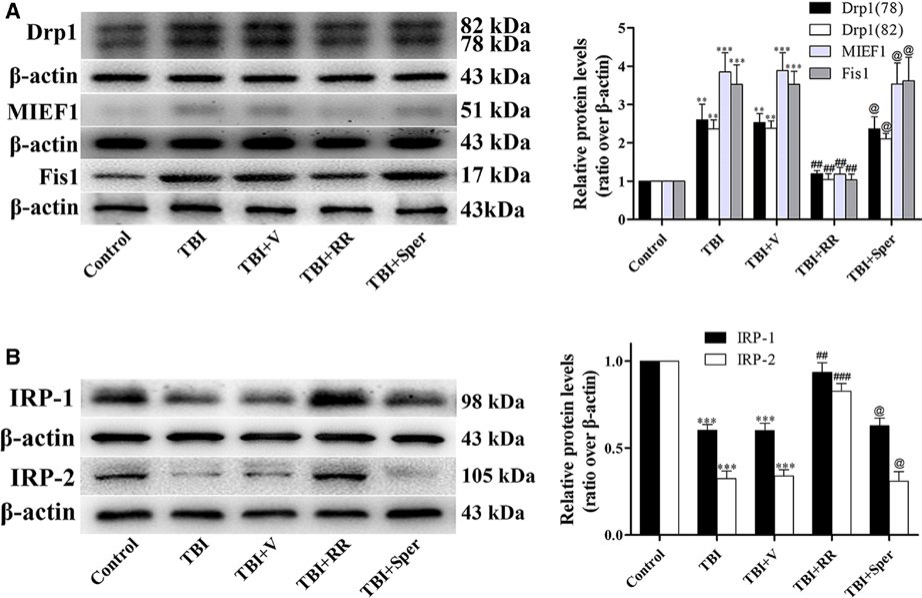
Traumatic brain injury (TBI)‐induced damage of mitochondria and disruption of iron homeostasis were improved by ruthenium red (RR) in primary cultured neurons. (A, B) RR treatment significantly decreased the expression of Drp1, MIEF1 and Fis1 (A) and increased the expression of IRP1/2 (B) after TBI, however, Sper treatment had no obvious effect on these proteins. Data are presented as mean ± SEM, n = 6 per group; **P < 0.01, ***P < 0.001 vs control group; ^##^ *P* < 0.01, ^###^ *P* < 0.001 vs TBI + vehicle group; ^@^ *P* > 0.05 vs TBI + vehicle group. β‐actin was used as a loading control

The neuroprotective effects of RR and Sper were also confirmed in primary cultured neurons. LDH release assay and TB staining were firstly conducted in neurons treated with RR or Sper. In LDH‐release assay, treatment of RR significantly decreased the percentage of damaged cells. Notably, Sper had no significant impact on cell death compared to the TBI + vehicle group (Figure 7A left). Similar results were found in TB staining (Figure 7A right). Then, to understand the effects of RR or Sper on neuron ROS levels, we used DCF‐DA fluorescence. Figure 7B showed that compared to the control neurons, the levels of ROS in the damaged neurons were significantly increased. When neurons were treated with RR, the levels of ROS were obviously decreased. Consistent with the results of LDH release assay and TB staining, treatment of Sper did not change the levels of ROS compared with the TBI + vehicle group (Figure 7B). Besides, the marker of apoptosis such as cleaved caspase‐3, the markers of mitochondrial damage such as Drp1, MIEF1 and Fis1 and the markers of iron accumulation such as IRP1/2 were analysed. We found that compared to the control neurons, cleaved caspase‐3, Drp1, MIEF1 and Fis1 were increased while IRP1/2 was decreased in the damaged neurons. RR instead of Sper treatment reversed these changes (Figures 7C and 8A and B). These results demonstrated that inhibition of MCU by RR increased cell viability, suppressed apoptosis and oxidative stress by inhibition of Ca^2+^ and iron accumulation in vitro.

## 4 DISCUSSION

In this study, we explored the role of MCU‐mediated Ca^2+^ and iron accumulation in TBI. We found that administration of RR, a blocker of MCU, provided neuroprotection in both in vivo and in vitro models of TBI. Interestingly, Sper, an opener of MCU, exhibited similar effects to RR in the in vivo model of TBI. The molecular mechanism of the neuroprotective effects of RR or Sper in TBI is by blockage of MCU to inhibit the accumulation of Ca^2+^ and iron.

The MCU is a key protein of the inner mitochondrial membrane which mediate the Ca^2+^ uptake into the matrix. MCU plays a crucial role in shaping of Ca^2+^ signalling and in control of aerobic metabolism.^6^ Under pathological conditions such as brain injury, MCU transports substantial Ca^2+^ to mitochondrion rapidly. Excessive mitochondrial Ca^2+^ uptake through MCU causes the dysfunction of mitochondrion, resulting in excitotoxicity that caused the generation of ROS, suppression of ATP and loss of MMP.^30^, ^31^ In addition, the generation of ROS could also modulate Ca^2+^ dynamics and raise Ca^2+^ surge.^32^ The interactions between Ca^2+^‐regulated generation of ROS and ROS‐induced increase of Ca^2+^ can form a feed forward, self‐amplified loop which further exacerbates cell damage far beyond simple Ca^2+^‐induced injury.^7^ Our results supported that Ca^2+^ accumulation after TBI stimulated the generation of ROS as a result of the defects in mitochondrial energy metabolism.

Since MCU is the main way to transport Ca^2+^ to mitochondrion, and the alteration of Ca^2+^ homeostasis is one of the key factors contributing to brain injury in TBI, blockage of MCU may decrease Ca^2+^ concentration and diminish the cell damage. Our results showed that inhibition of MCU by RR or Sper could significantly suppress neuron death and apoptosis via decreasing the mitochondrial Ca^2+^ concentration, indicating that excessive mitochondrial Ca^2+^ uptake by MCU could be responsible for TBI‐induced neuronal degeneration.

Iron is necessary for cell growth and differentiation in multiple organs particularly in the brain because of its role as a constituent of molecules generating mitochondrial energy.^33^ Although iron plays an important role in CNS, abnormally high levels of iron in brain may lead to cognitive and motor deficits.^34^, ^35^ The mechanisms of iron overload‐induced brain disorders are related to the dysfunctions of mitochondrion.^36^ Iron enters the mitochondrion, reacts with lipids to generate alkoxy and peroxy radicals and with hydrogen peroxide to form hydroxyl radicals, causing the generation of ROS.^37^ ROS then leads to the depolarization of MMP and subsequently damages mitochondrion.^38^ Therefore, iron is a potential target for treatment of brain trauma. However, the transmembrane pathway of iron entery into mitochondrion is still unclear. There are studies showing that MCU could facilitate mitochondrial iron uptake.^15^, ^39^ Under normal conditions, Ca^2+^ enters the mitochondrion through MCU. However, other anions such as Mn^2+^ and Sr^2+^ could also enter the mitochondrion via MCU because of the equal positive charges.^40^ As iron (Fe^2+^) and Ca^2+^ also have equal positive charges, it may be the reason that iron could use MCU as a pathway to enter into mitochondrion. Consistently, our study showed that the inhibition of MCU obviously attenuated iron overload. As both Ca^2+^ and iron could translocate into mitochondrion through MCU, suppression of Ca^2+^ and iron accumulation by blockage of MCU may stabilize mitochondrion. As a result, ROS generation reduced, energy supply increased and neuronal cell death and apoptosis decreased.

Although Sper has been proposed as an antioxidant in physiological concentrations,^41^ the protective effects of Sper observed in our TBI models differ from its role in other models of brain injury.^22^, ^42^ In these studies, Sper was shown to promote neuronal cell death and apoptosis by activating MCU. However, our results were consistent with the results demonstrating that Sper was involved in the control of Ca^2+^ and iron homeostasis as well as antioxidation.^43^, ^44^ The difference of pro‐oxidant or antioxidant effects of Sper depends on which environment cells are in. Under normal conditions, Sper is bidirectionally transported through the inner membrane by cycling, in which efflux and influx are driven by pH and electrical gradients respectively.^45^ However, TBI‐induced mitochondrial damage can cause the inner membrane’s partial permeability, which further leads to the movement of ions along the concentration gradient. Thus, the Sper cycling terminates, the excessive Sper in mitochondria could prevent the Ca^2+^ and iron accumulation in mitochondrion, and eventually protect mitochondrion against TBI. Interestingly, although Sper provided neuroprotection in the in vivo model of TBI, it had no neuroprotective effects in the in vitro model of TBI. The reasons were unclear，but we would clarify the underlying mechanisms in our future studies.

Our study has some limitations. Firstly, recent evidence has suggested that spermine could improve brain function and protect neurons from ischaemic injury by enhancing autophagic flux.^46^, ^47^ Moreover, induction of autophagic flux in immune cells by spermine may also reduce brain injury by suppressing inflammation.^48^, ^49^ Given that spermine activates rather than suppresses MCU activity, these mechanisms may explain the neuroprotective effects of spermine in the in vivo TBI model that is characterized by inflammation and the neuroprotective failure in the in vitro TBI model which is not. Secondly, RR is a general inhibitor of sodium and calcium channels,^50^ it also blocks ryanodine receptors located in the sarcoplasmic/endoplasmic reticulum that may promote cell death.^51^, ^52^ Thus, RR is not a selective MCU inhibitor and may have protected neurons by blocking excessive plasma membrane calcium and sodium channel activities as well as the ryanodine receptor in addition to the MCU. Thirdly, RR has a strong positive charge and does not cross the blood‐brain barrier.^54^ Therefore, RR may be unlikely to reach brain concentrations necessary for neuroprotection following systemic administration. This suggests that RR may act on peripheral sites to reduce brain injury such as vascular endothelial cells,^55^ so reduced mitochondrial calcium content in mice subjected to TBI by RR may be secondary to decreased brain injury. However, these speculations are obtained from previous literatures and whether theses speculations are correct is unclear, we will clarify them in our future studies.

In conclusion, our study indicated that inhibition of MCU or prevention of Ca^2+^ and iron accumulation could exert neuroprotection against TBI by combating mitochondria damage, oxidative stress, neuronal cell death and apoptosis. These results make MCU a potential therapeutic target in TBI treatment in the future.

## CONFLICT OF INTEREST

The authors declare no potential conflicts of interest.
